# A Nomogram of Fetal Nasal Bone Length at 16-24 Weeks of Gestation in North Indian Pregnant Women: A Prospective Study

**DOI:** 10.7759/cureus.98575

**Published:** 2025-12-06

**Authors:** Ashok K Verma, Arpit Singh, Pavika Lal, Renu Gupta, Poonam S Gambhir, Neena Gupta, Divya Dwivedi, Deepak Anand

**Affiliations:** 1 Radiodiagnosis, Ganesh Shankar Vidyarthi Memorial (GVSM) Medical College, Kanpur, IND; 2 Obstetrics and Gynaecology, Ganesh Shankar Vidyarthi Memorial (GVSM) Medical College, Kanpur, IND; 3 Medical Genetics, Vardaan Genetic and Diagnostic Centre, Kanpur, IND; 4 Social and Preventive Medicine, Ganesh Shankar Vidyarthi Memorial (GVSM) Medical College, Kanpur, IND

**Keywords:** chromosomal abnormalities, nasal bone length, prenatal screening, second trimester ultrasound, trisomy 21

## Abstract

Introduction

Fetal nasal bone length (NBL) is a crucial sonographic marker in prenatal screening for chromosomal abnormalities, particularly trisomy 21. However, its diagnostic accuracy is influenced by ethnic variations, and normative data for North Indian populations remain scarce.

Objective

This study aimed to establish a gestational age-specific nomogram for fetal NBL between 16+1 and 24 weeks of gestation in North Indian pregnant females, and to evaluate its correlation with standard fetal biometric parameters.

Methods

A prospective observational study was conducted on 438 structurally and chromosomally normal North Indian pregnant females. Fetal NBL was measured using mid-sagittal ultrasound imaging, and participants were stratified into two gestational age groups, A and B. Group A includes 16-20 weeks of gestational age, and Group B includes more than 20 to 24 weeks of gestational age. Percentile distributions were calculated, and correlations between fetal NBL and other biometric parameters (biparietal diameter (BPD), head circumference (HC), abdominal circumference (AC), femur length (FL)) were assessed.

Results

Mean fetal NBL increased significantly with gestational age (4.56 mm in Group A vs. 5.74 mm in Group B; p < 0.001). Strong positive correlations were found between fetal NBL and BPD (r = 0.926), HC (r = 0.903), and AC (r = 0.874), while a moderate correlation was noted with FL (r = 0.402). The fifth-percentile thresholds for fetal NBL were 3.70 mm (Group A) and 5.10 mm (Group B).

Conclusion

This study provides a population-specific nomogram for fetal NBL in North Indian pregnancies during the second trimester. The findings underscore the importance of using ethnicity-specific reference values to improve the accuracy of prenatal aneuploidy screening and to reduce false-positive diagnoses.

## Introduction

Genetic sonography serves as a vital technique in prenatal evaluation, providing detailed imaging that aids in the early detection of potential fetal abnormalities. The first report of the association between the ultrasonographic absence of the fetal nasal bone and Down syndrome was recently published [1]. The most often used parameters in the second trimester of pregnancy are the biparietal diameter (BPD), head circumference (HC), abdominal circumference (AC), and femur length (FL). These parameters are considered the “gold standard,” as they collectively assess gestational age with the highest degree of accuracy. Reference ranges for these fetal biometric parameters by ultrasound were initially reported by Hadlock et al. [2-4] in populations of developed countries. If not assessed properly, the nasal bone may appear shorter than its actual length or may even be misinterpreted as absent. The accuracy of nasal bone assessment can be affected by various factors, such as the resolution of the ultrasound machine, the skill and experience of the sonographer, maternal body habitus, the position of the fetus, and the stage of gestation. The various biometric parameters used for fetal growth assessment are BPD, HC, AC, and FL, according to International Society of Ultrasound in Obstetrics and Gynecology (ISUOG) guidelines [5]. Ethnic background plays a significant role in determining the morphometric features of the nasal bone. Reference ranges for fetal nasal bone length (NBL) have been established for populations of Caucasian, African-American, and South American descent. However, data specific to populations in the Indian subcontinent remain limited. Because of substantial ethnic variation in facial symmetry, applying reference standards from other populations may result in normal Indian fetuses being incorrectly identified as at risk for aneuploidy. It is therefore crucial to develop population-specific reference ranges for NBL measurements to enhance diagnostic precision and minimize the risk of misinterpretation in prenatal screening [6].

Limited data are available in the North Indian population regarding NBL and its correlation with BPD, FL, HC, and AC. To fill this gap, the present study was conducted. The objective of the study is to contribute to the development of a nomogram that can be used in the second trimester for diagnosing fetal nasal bone hypoplasia as a means of predicting and screening aneuploidy cases in the North Indian population. 

## Materials and methods

It was a prospective observational study conducted in the Department of Radiodiagnosis, in collaboration with the Obstetrics and Gynaecology Department, Ganesh Shankar Vidyarthi Memorial (GVSM) Medical College, Kanpur, India, with the help of a high-resolution ultrasound machine. Data were collected from May 2024 to May 2025. Ethical approval was obtained from the ethics committee of our institute before undertaking the study (Ref. No. EC/225/May/2024). 

All pregnant females with a singleton pregnancy from 16 to 24 weeks of gestational age, attending the Outpatient Department (OPD) and Inpatient Department (IPD) of the hospital, were enrolled in the study. All of the patients included were residents of North Indian states, namely Uttar Pradesh, Bihar, and Delhi. The study was conducted on 438 participants.

Participants were categorized into Groups A and B. Group A included pregnant females with a singleton pregnancy from 16 to 20 weeks of gestational age, and Group B included females with a singleton pregnancy from >20 to 24 weeks of gestational age. Pregnant females with <16 weeks or >24 weeks of gestation, early fetal growth restriction, fetal anatomic abnormalities, multiple pregnancies, fetal death in utero, a fetus with an absent nasal bone, and cases of non-North Indian origin were excluded from the study.

Gestational age was calculated using ultrasound, following the Hadlock method. The USG machines used were the Samsung RS80A (Samsung Medison Co., Ltd., Seoul, South Korea) and the GE Versana Balance Touch (GE HealthCare, Chicago, IL, USA), with a curvilinear probe of frequency range 2-5 MHz. The operator had 18 years of work experience. 

**Figure 1:**
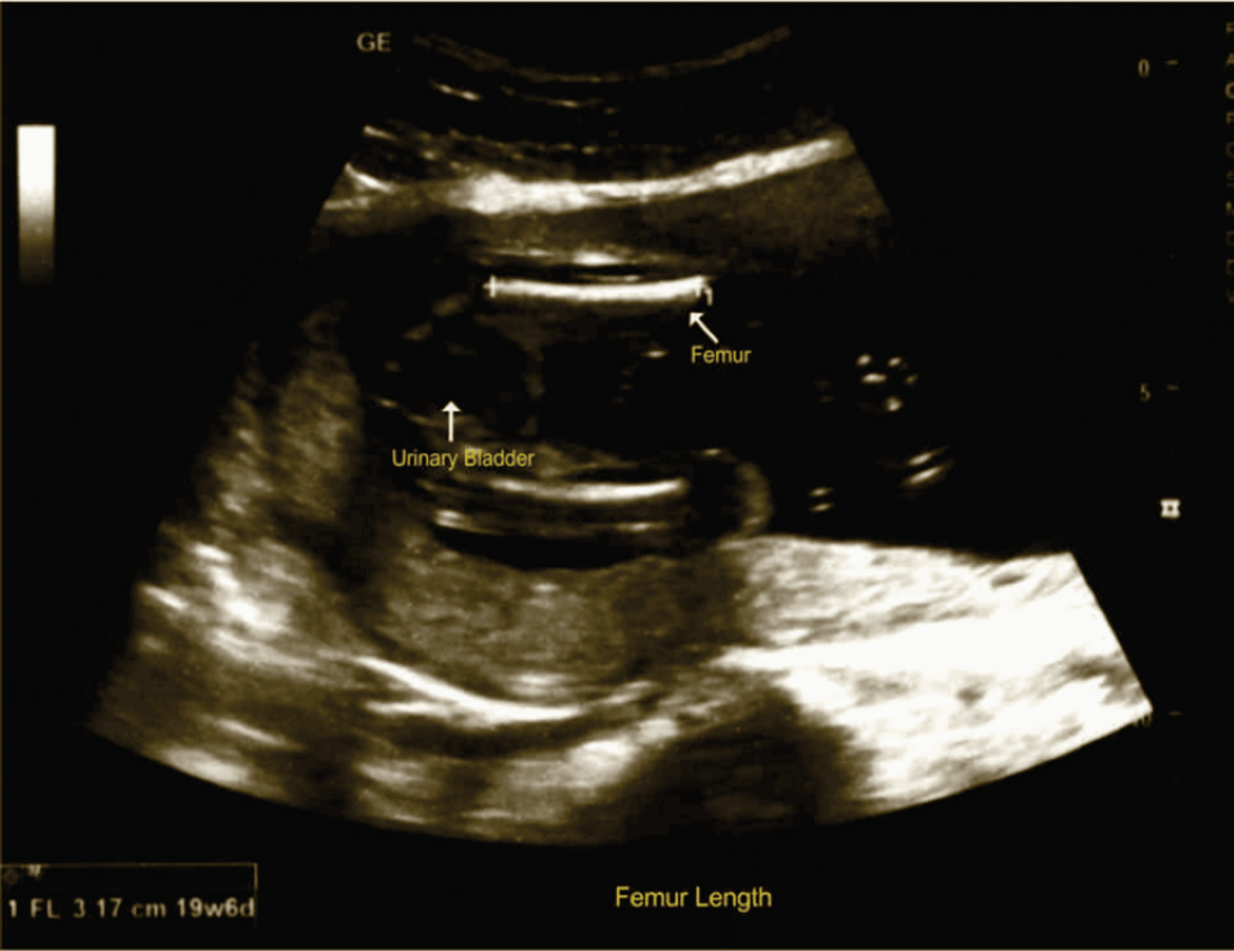
Fetal femur length measurement

Figure 1 shows an ultrasonographic measurement of fetal FL; the scan was performed according to ISUOG guidelines [5].

**Figure 2:**
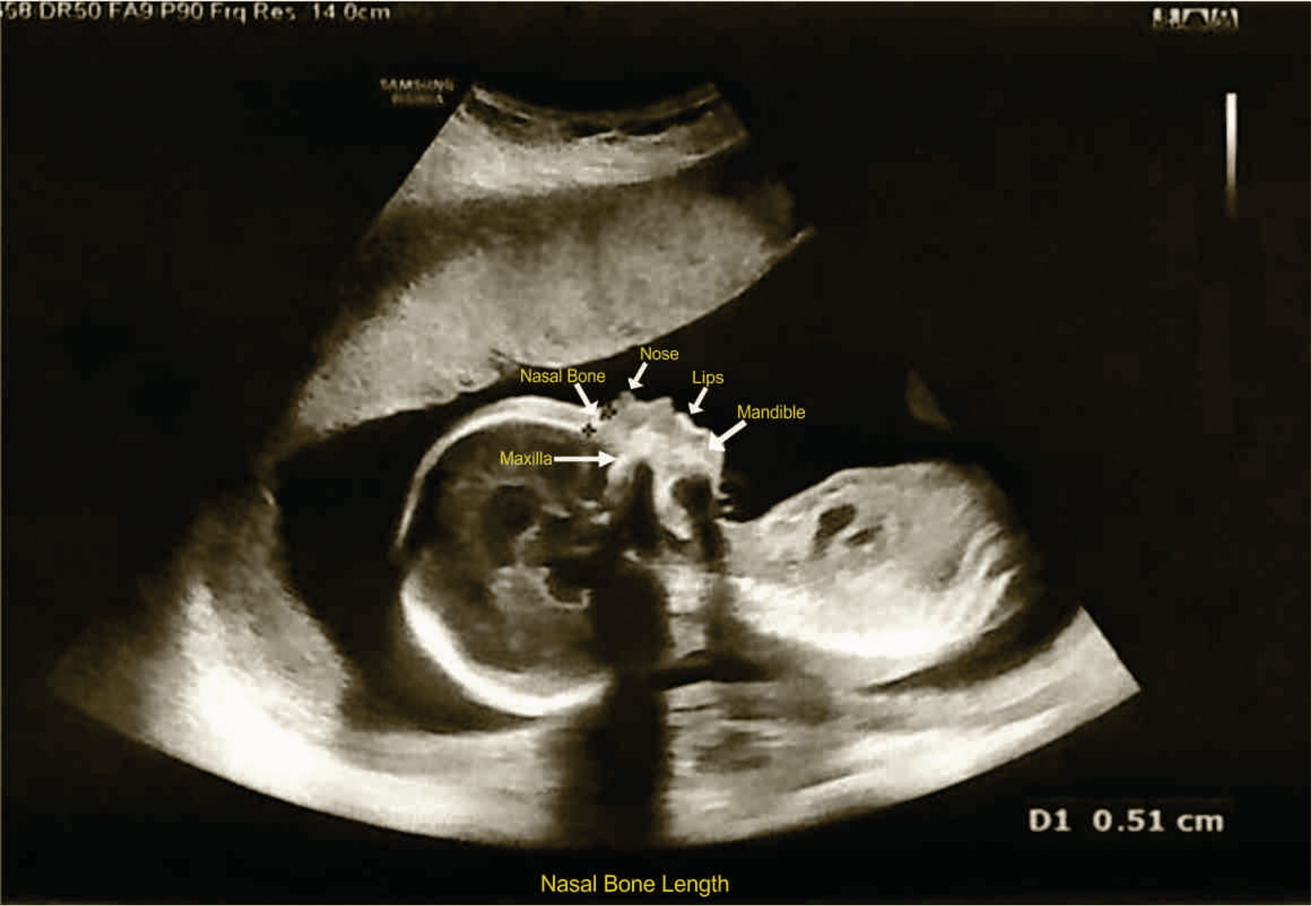
Fetal nasal bone length

Figure 2 shows the measurement of the fetal nasal bone, performed by a single operator on a midsagittal view of the fetal head, identifying the nasal bone, lips, maxilla, and mandible, with an angle between the insonation beam and the nasal bone axis close to 45° or 133°, following the method described by Sonek and Nicolaides [1]. The maximum length was measured in millimeters, and three independent images were obtained; the average of all three measurements was taken for each patient. The fetal growth parameters, including BPD, HC, AC, and FL, were assessed for each fetus.

**Figure 3:**
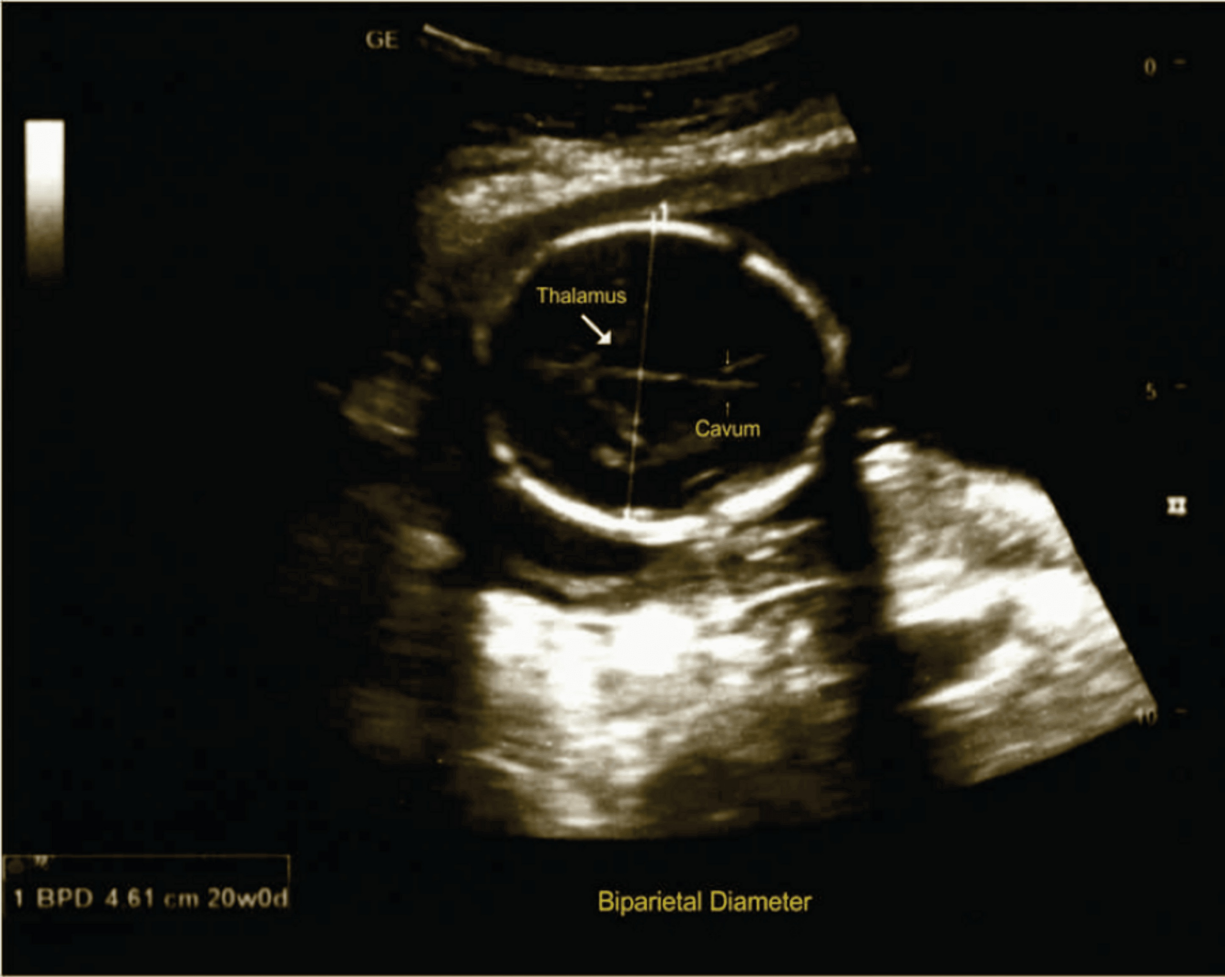
Biparietal diameter measurement

Figure 3 shows the ultrasonographic measurement of BPD; the scans were performed according to ISUOG guidelines [5].

**Figure 4:**
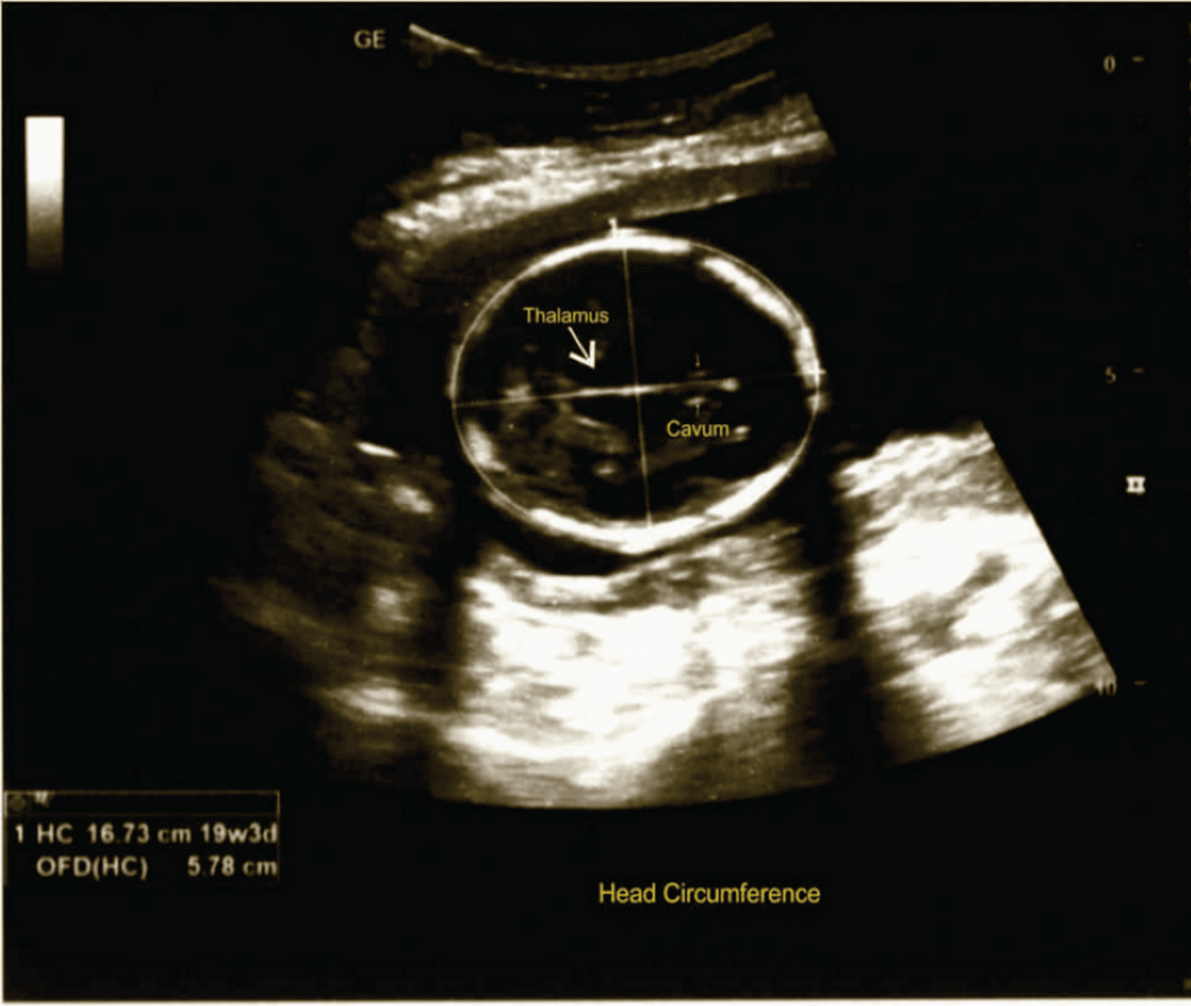
Head circumference measurement

Figure 4 shows the ultrasonographic measurement of fetal HC; the measurement was performed according to ISUOG guidelines [5].

**Figure 5:**
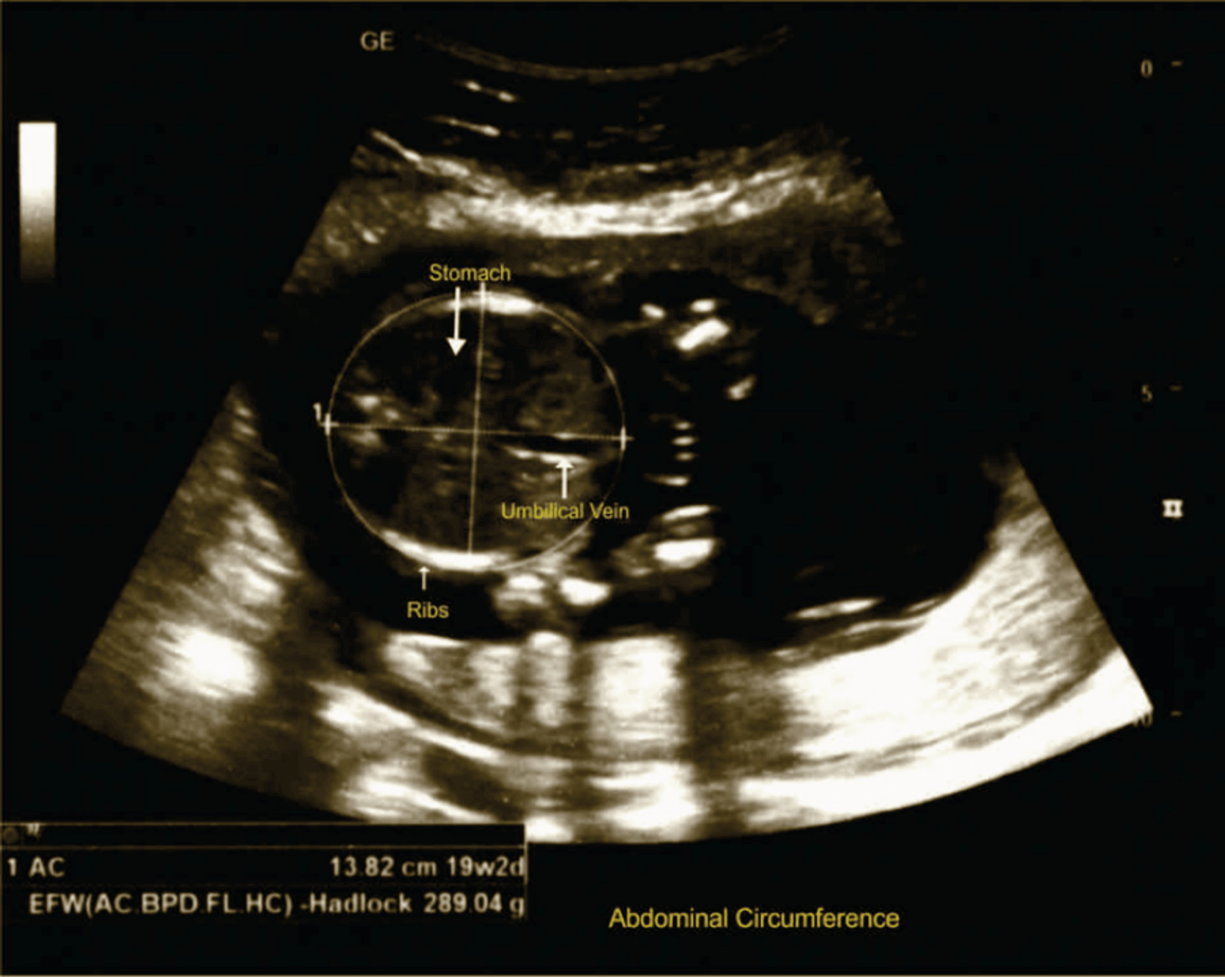
Abdominal circumference measurement

Figure 5 shows the measurement of fetal AC; the scan was performed according to ISUOG guidelines. 

Statistical analysis was performed using IBM SPSS Statistics for Windows, Version 26 (Released 2017; IBM Corp., Armonk, NY, USA). Pearson’s correlation and linear regression analysis were used to analyze the relationship between the fetal NBL and BPD, HC, AC, and FL. A p-value <0.05 was considered statistically significant.

## Results

The present study analyzed a group of 438 North Indian pregnant females undergoing second-trimester fetal ultrasound between 16-20 and >20-24 completed weeks of gestation. All ultrasonographic scans were successfully performed, and data for all growth parameters were appropriately obtained for all 438 subjects. The mean age of Group A participants and Group B participants was 26.78 years and 27.02 years, respectively (Table 1). 

**Table 1:** Mean age of participants

Participants	Mean age in years
Group A	26.78
Group B	27.02

Table 2 depicts the correlation between NBL and fetal biometric parameters. The results show that, regarding the BPD, a highly significant correlation with NBL was found. Regarding HC, a highly significant correlation was also found with NBL. Likewise, AC and FL also show a highly significant correlation with NBL.

**Table 2:** Correlation between nasal bone length and fetal biometric parameters Level of significance: p < 0.05

Variable	Pearson correlation (r)	p-value
Biparietal Diameter (cm)	0.926	< 0.001
Head Circumference (cm)	0.903	< 0.001
Abdominal Circumference (cm)	0.874	< 0.001
Femur Length (cm)	0.402	< 0.001

Fetal biometric parameters assessed in both groups, including BPD, HC, AC, FL, and NBL, are presented in Table 3. An independent t-test was applied to evaluate statistical significance. Regarding BPD, HC, AC, FL, and NBL, a statistically significant difference was found between Groups A and B.

**Table 3:** Group-wise comparison of fetal biometric parameters Level of significance: p < 0.05

Parameter	Group	N	Mean	SD	t-test value	p-value
Biparietal Diameter (cm)	A	252	4.27	0.41	25.53	<0.001
	B	186	5.29	0.42		
Head Circumference (cm)	A	252	15.63	1.78	24.01	<0.001
	B	186	19.46	1.54		
Abdominal Circumference (cm)	A	252	13.3	1.73	22.16	<0.001
	B	186	16.71	1.48		
Femur Length (cm)	A	252	2.99	1.22	9.21	<0.001
	B	186	3.75	0.39		
Nasal Bone Length (mm)	A	252	4.56	0.51	24.85	<0.001
	B	186	5.74	0.47		

The nomogram of the fetal NBL from 16 to 24 weeks of gestation was established in the present study and is presented in Table 4. The median NBL values increased from 3.7 mm at 16 to 16 weeks + 6 days to 6.40 mm at 23-24 weeks.

**Table 4:** Nomogram of the fetal nasal bone length in millimeters obtained from the present study

Gestational age (weeks-weeks+days)	N = 438	5th percentile	50th percentile	95th percentile
16-16+6	33	3.56	3.70	3.94
17-17+6	40	3.80	4.10	4.40
18-18+6	62	4.20	4.60	4.90
19-19+6	104	4.51	4.90	5.20
20-20+6	88	5.10	5.30	5.60
21-21+6	35	5.20	5.60	5.93
22-22+6	35	5.70	5.95	6.23
23-24	41	6.20	6.40	6.60

**Figure 6:**
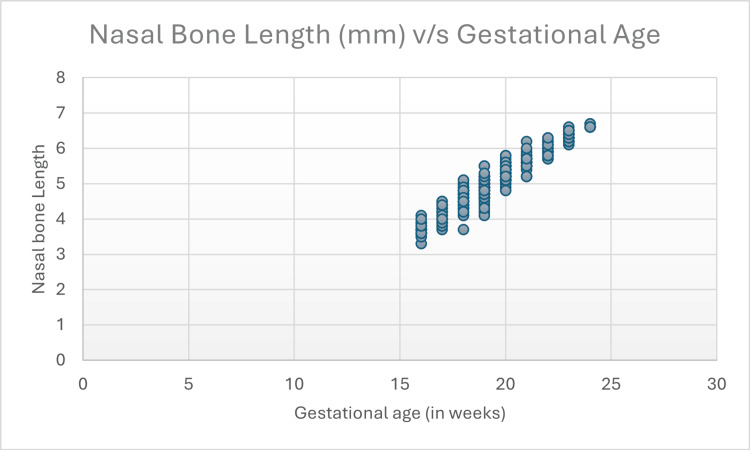
Scatter plot of gestational age versus nasal bone length

Figure 6 shows the NBL in millimeters versus gestational age. The figure demonstrates an increase in NBL with increasing gestational age. 

## Discussion

Our study is an attempt to establish a nomogram of fetal NBL and its correlation with other biometric parameters, such as BPD, HC, AC, and FL, in the second trimester in the North Indian population. Table 5 shows a comparison of median NBL values in different studies. It shows that the fetal NBL in the North Indian population is comparable to that of the South Indian population, but it varies from other ethnicities.

**Table 5:** Comparison of median NBL values of different studies *Represents the 23-24 completed weeks' result of the present study NBL, nasal bone length; ND, not determined

Gestational age (weeks)	Caucasian and African-American [7]	Indian [6]	North Indian [8]	South Indian [9]	Korean population [10]	São Paulo, Brazil [11]	Japanese population [12]	Chinese population [13]	Present study
16-16+6	4.7	4.1	3.5	3.5	3.0	5.9	3.5	4.1	3.70
17-17+6	5.3	4.5	4.2	4.2	3.5	6.2	4.5	4.6	4.10
18-18+6	5.7	4.6	4.5	4.6	3.8	6.5	4.9	5.0	4.60
19-19+6	6.3	5.2	4.8	4.9	4.8	6.8	5.2	5.6	4.90
20-20+6	6.7	5.4	5.0	5.3	5.4	7.0	5.8	5.8	5.30
21-21+6	7.1	5.7	5.2	5.7	5.7	7.3	5.7	6.2	5.60
22-22+6	7.5	6.5	5.8	6.0	6.0	7.6	6.6	6.7	5.95
23-23+6	7.9	6.5	ND	ND	6.3	7.8	ND	ND	6.40*
24-24+6	8.3	6.7	ND	ND	7.0	8.0	ND	ND	

Papasozomenou et al. revealed that different ethnic groups have considerably varying normal ranges for the length of the fetal nasal bone in the second trimester. Therefore, rather than using an international model, different ethnic nomograms of fetal NBL in the second trimester should be employed in a particular group [14]. Gamez et al. [15] reported a longer nasal bone in Caucasians than that reported by Jung et al. in the Korean population [10]. Biswas et al. also reported that comparison of nomograms of NBL from different ethnic origins revealed that the NBL values in their study were significantly lower than those in Caucasian populations, and that an indigenous reference range for fetal NBL is better suited for use in the Indian population [6]. Likewise, similar to the findings of our study, Sharma et al. reported a significant increase in NBL with gestational age, BPD, and FL, and found the NBL to be shorter compared with other ethnicities [16]. Likewise, similar to the findings of our study, a study conducted by Kashikar and Lakhkar also revealed that the mean NBL increased linearly with advancing BPD [17].

Yanik et al. conducted a study in Turkey and likewise reported that fetal NBL correlated significantly with gestational age, BPD, and FL measurements [18]. Yayla et al. also revealed that measurement of the NBL during gestation shows a linear growth pattern according to gestational week, BPD, and FL [19]. A Korean study also showed that biometry profiles increase linearly as a function of BPD [20]. Fetal NBL and BPD are linearly related in the second trimester and are reported to be shorter in the Korean population than in Caucasians and African Americans. This finding was similar to our study, in which an increase in NBL had a positive correlation with BPD. Similar to our study, a study conducted by Goynumer et al. on the Turkish population revealed a significant positive correlation between fetal NBL and gestational age and between fetal NBL and BPD [21]. A study conducted by Prabhu et al. depicted a significant statistical correlation between NBL and BPD, HC, AC, and FL [22].

Many researchers, such as Sonek and Nicolaides and Bunduki et al., have published multiple reports on the normal range of NBL throughout gestation [1,11]. Many studies have highlighted the importance of measuring fetal NBL for prenatal screening. Küpeli et al. established an NBL nomogram in healthy fetuses between 18+0 and 23+6 gestational weeks for use in the diagnosis of nasal bone hypoplasia in prenatal screening [23]. Prakash Jain et al. highlighted the importance of nasal bone evaluation in the second trimester of pregnancy to detect fetuses with Down syndrome. Since NBL increases linearly with gestational age, it aids in detecting hypoplastic nasal bone at different gestational ages [24]. Tran et al. conducted a study to evaluate the utility of the fetal BPD-to-NBL ratio in the second trimester as a screening tool for trisomy 21 and concluded that combining BPD/NBL with other sonographic markers may improve trisomy 21 detection [25]. Vikraman et al. also revealed that the mean NBL values of the Indian population are lower than those of the Caucasian population [26]. Mogra et al. revealed that there is no significant difference in NBL between Caucasian and Asian populations, and that it is reasonable to use criteria established in a Caucasian population to define the characteristics of an absent or short nasal bone in Asian fetuses [27].

This study was conducted in only one medical college on a limited sample size; more accurate reference values can be obtained by conducting a multicentric study on a larger sample size in northern India. 

## Conclusions

Our study showed that the range of NBL measurement varied with region as well as ethnicity, highlighting the fact that standardization of NBL is essential to exclude hypoplasia. Nasal bone hypoplasia has emerged as one of the strongest morphological markers of trisomy 21, and the nomogram obtained from this study can be integrated into prenatal screening protocols, as it will be useful in reducing false positives in Down syndrome risk estimation. Strong positive correlations were observed between NBL and other biometric parameters, particularly BPD, HC, AC, and FL, all of which are statistically significant. These findings affirm that NBL development progresses synchronously with overall fetal somatic growth. 
